# Neuromuscular synergy characteristics of Tai Chi leg stirrup movements: optimal coordination patterns throughout various phases

**DOI:** 10.3389/fbioe.2024.1482793

**Published:** 2024-10-23

**Authors:** Xiaopei Zhang, Mengyao Jia, Yong Ke, Jihe Zhou

**Affiliations:** ^1^ School of Tennis, Wuhan Sports University, Wuhan, China; ^2^ Engineering Research Center of Sports Health Intelligent Equipment of Hubei Province, Wuhan Sports University, Wuhan, China; ^3^ Key Laboratory of Sports Engineering of General Administration of Sport of China, Wuhan Sports University, Wuhan, China; ^4^ School of Sports Medicine and Health, Chengdu Sport University, Chengdu, China

**Keywords:** Tai Chi, leg stirrup movements, muscle synergy, dynamic stability, EMG

## Abstract

**Objective:**

To investigate the neuromuscular activity characteristics of Tai Chi athletes and identify optimal muscle synergy patterns.

**Method:**

Data were collected from 12 elite Tai Chi athletes using a Vicon motion capture system, a Kistler 3D force plate, and a Noraxon surface electromyography system. Muscle synergy patterns were extracted using Non-negative Matrix Factorization.

**Results:**

Four muscle synergy patterns were identified in each of the three phases of the leg stirrup movement, with the optimal synergy pattern for each phase determined as follows: knee lift phase: rectus femoris and vastus lateralis of the right leg; extension phase: rectus femoris, vastus lateralis, biceps femoris, and medial gastrocnemius of the right leg; recovery phase: rectus femoris, vastus lateralis, and medial gastrocnemius of the right leg. These patterns explain the muscle coordination activities for each phase.

**Conclusion:**

This study identified the optimal muscle synergy patterns for each phase, supporting the fluidity and force generation of the leg stirrup movement. This provides Tai Chi athletes with a more efficient way to exert strength and maintain balance.

## Introduction

The Tai Chi Leg Stirrup Movement is a special type of movement (Shen et al., 2018) and one of the more difficult leg techniques (Huang et al., 2009), requiring high demands on the neuromuscular system. They involve precise control of strength, coordination, and balance. To complete the leg kick, one leg is supported with a slight bend, while the other leg is lifted with a bent knee and toes hooked, Stirrup out to the side front. Key points include lifting the knee as high as possible, hooking the toes, directing force to the heel, keeping the upper body upright, and maintaining stable balance (Ma and Jennings, 2021). This movement requires athletes to maintain overall balance while quickly and accurately mobilizing the muscles. Therefore, coordination ability of the neuromuscular system plays a critical role in this process.

Muscle synergy refers to the effective coordination of multiple muscle groups to produce smooth and powerful movements (Sheng et al., 2021; Overduin et al., 2015; Chang et al., 2014). Muscle synergy analysis methods can be used to identify muscle coordination strategies in different populations (Ao et al., 2022; Wang and Liu, 2022). Muscle synergy is obtained by extracting surface electromyography (sEMG) signals and performing blind source separation to obtain a set of muscle combinations with inherent spatiotemporal activation characteristics, thus defining the contribution of each synergy to muscle activation (Pellegrino et al., 2020; Turpin et al., 2011). Although the neurogenic origin of the muscle synergy theory is still debated and not fully proven, it has been shown to be effective in studying human motor control mechanisms and revealing potential ways to optimize athletic performance. Muscle synergy methods have been widely applied in various sports (Severini and Zych, 2020), particularly in the study of motor techniques, strength balance, exercise efficiency, and injury prevention. For example, Wu et al. (2017) found differences in the electromyographic characteristics of hip extensor muscles in female short-distance speed skaters during left and right single-step ice pushes, indicating technical differences between legs. Improving the movement structure helps balance the strength of both legs and addresses the problem of divergent movements in the later phases of a race. Wang et al. (2020) found asynchronous changes in lower limb muscle synergy during a 30-s all-out cycling exercise, leading to decreased efficiency in quadriceps-gastrocnemius force transmission and increased co-contraction ratio of ankle antagonists. They suggested training to increase the speed endurance of the related muscles to delay and reduce muscle fatigue. Zhang et al. (2021) used a time synergy model to identify four muscle synergies in national female shooting athletes corresponding to different technical functional tasks during archery. Cui et al. (2023) found that as walking speed increased, the connection strength between local muscles increased, with different muscle synergy patterns and functional networks observed at different speeds. Tai Chi exercise can improve lower limb muscle strength and body balance. Long-term Tai Chi practice enhances muscle activation around the knee joint and strengthens coordination between muscle groups, helping stabilize the joint (Yu et al., 2022). However, research on how the central nervous system controls and coordinates muscle organization in Tai Chi remains limited.

Therefore, this study aimed to explore the neuromuscular activity characteristics of Tai Chi athletes performing Leg Stirrup Movements and seeking optimal muscle synergy patterns at various phases. This research provides scientific support for Tai Chi training and performance and offers correct references for beginners practicing Leg Stirrup Movements.

## Materials and methods

### Research subjects

In this study, we used G Power 3.1.9.2 to calculate the required sample size with an effect size of 0.75. Under the conditions of α = 0.05 and a statistical power of 95%, 12 participants were needed (Kang, 2021). This study recruited 12 high-level Tai Chi athletes from Wuhan Sports University, aged between 16 and 28 years. Among them, there were 6 national master-level athletes, 2 first-level athletes, and 4 second-level athletes. Their average age was 18.83 ± 3.32 years, average height was 173.08 ± 5.58 cm, average weight was 63.33 ± 6.56 kg, and average years of martial arts experience was 9.08 ± 4.78 years. All participants had good physical fitness and athletic ability, with no history of injuries or diseases in the past year. Before the experiment, they thoroughly read the experimental plan, understood the purpose, methods, testing procedures, and potential risks of the study, and signed informed consent forms. This study was approved by the Medical Ethics Committee of Wuhan Sports University (Approval number: 2024048). Confirming that all experiments were performed in accordance with relevant guidelines and regulations.

## Research methods

### Data collection

The experiment was conducted at the Key Laboratory of Sports Engineering, General Administration of Sports, Wuhan Sports University. A Vicon motion capture system (T40, Vicon, UK, with a sampling frequency of 200 Hz), a Kistler 3D force platform (9260AA6, Switzerland, with a sampling frequency of 1500 Hz), and a Noraxon surface electromyography (EMG) system were used to synchronously collect motion capture data, force data, and surface EMG data of Tai Chi Leg Stirrup Movements.

A modified version of the Visual 3D default full-body model with 38 marker points was used. The specific marker placement was as follows: left and right forehead (LHAD, RHAD), left and right rear head (LFAD, RFAD), clavicle (CLAV), seventh cervical vertebra (C7), sternum (STRN), 10th thoracic vertebra (T10), left and right anterior superior iliac spines (LASIS, RASIS), left and right iliac crests (LICST, RICST), left and right posterior superior iliac spines (LPSIS, RPSIS), left and right greater trochanters (LTROC, RTROC), left and right thigh tracking points (LTH1, LTH2, RTH1, RTH2), left and right knee medial and lateral epicondyles (LMEP, RMEP, LLEP, RLEP), left and right shank tracking points (LSK1, LSK2, RSK1, RSK2), left and right ankle medial and lateral malleoli (LMME, RMME, LLME, RLME), left and right heels (LHEEL, RHEEL), left and right first metatarsals (LHM1, RHM1), and left and right fifth metatarsals (LHM5, RHM5).

Since the leg push is mainly designed to extend the flexion and extension of the hip, knee and ankle joints, this study selected 12 muscles of the left and right legs: rectus femoris (RF), lateralis femoris (VL), tibialis anterior (TA), biceps femoris (BF), medial head of gastrocnemius (MG) and gluteus maximus (GMAX). The knee extensor includes the rectus femoris and the lateral femoris, which are mainly responsible for knee extension, and the knee flexor includes the biceps femoris. The ankle plantar flexor group includes the medial head of gastrocnemius muscle, and the ankle dorsiflexor group includes the tibialis anterior muscle. The hip flexor group includes the gluteus maximus.

### Testing procedure

The experimental procedure and precautions were explained to the subjects, ensuring that they signed the informed consent form after full understanding. The subjects’ basic information was recorded, and key morphological indicators, such as trunk, upper arm, forearm, thigh, shank, and foot lengths, were measured. Based on the Visual 3D full-body model, 38 marker points were securely attached to the subjects’ bodies. To reduce errors, the subjects wore lab-specific tight clothing, and the same tester placed the markers each time. After placement, each sensor was checked for secure attachment and the sensors on the measured muscles were verified to be correctly connected to the corresponding channels. The skin surface of the measured muscles was cleaned with 75% alcohol swabs and shaved to ensure successful EMG signal collection.

**FIGURE 1:**
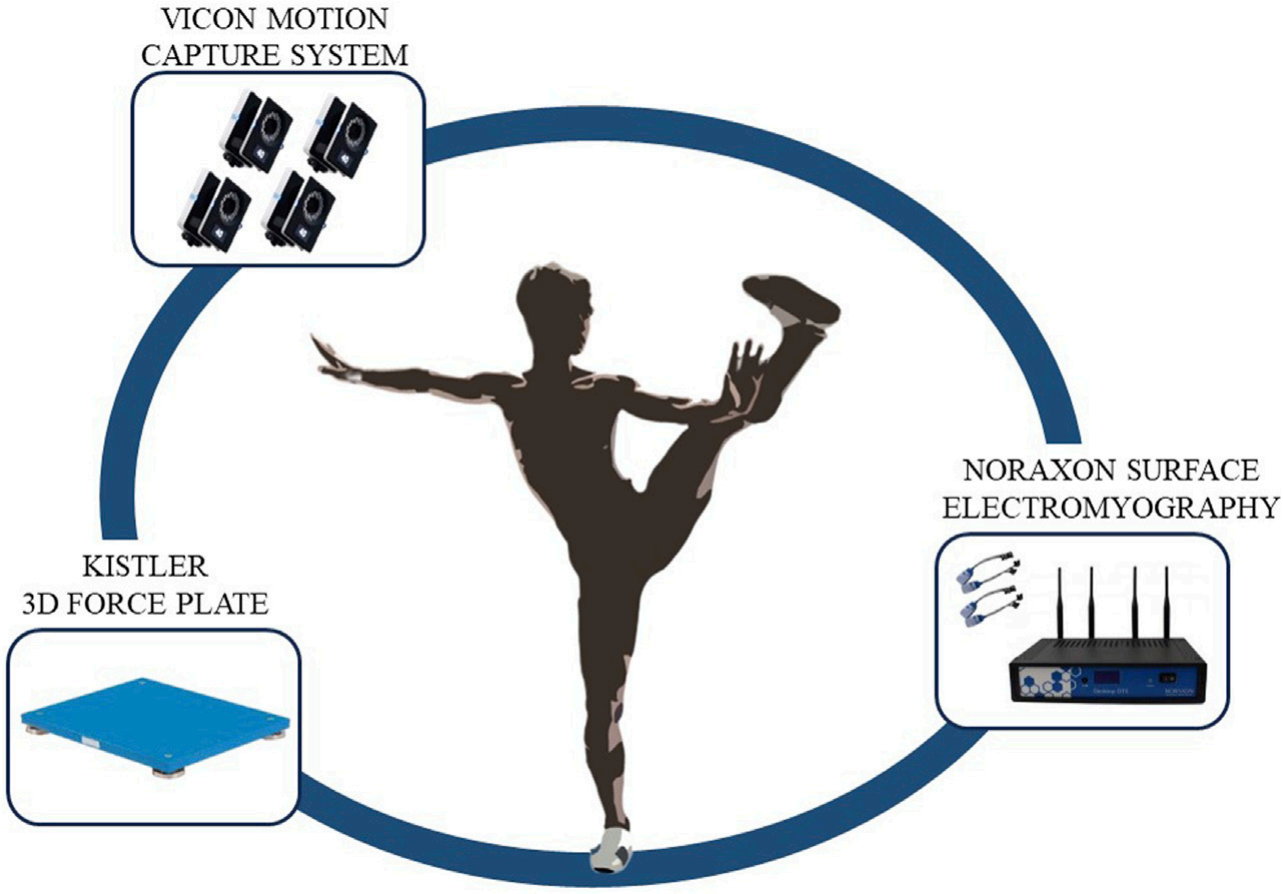
Schematic diagram of experimental testing.

Before the experiment began, static data were collected once, followed by a 5-min warm-up to prevent muscle strain during the test. During formal data collection, the entire process was videotaped. The testers guided the subjects to position themselves in the motion capture area with one foot on a force platform. The subjects performed the Tai Chi Leg Stirrup Movements based on the tester’s commands, repeating the action five times. A Tai Chi coach checked whether the subjects’ movements met standard requirements. The test scenario is shown in Figure 1.

### Data processing

Import the raw kinematic data from the Vicon motion capture system into Visual 3D for inverse dynamics calculations. Use the calculated center of mass displacement to further compute the velocity of the center of gravity (VCG) using Matlab R2022a, as shown in Equation 1:

$$VCG=\frac{d\left(t_{i+1}\right)\;‐\;d\left(t_{i}\right)}{t_{i+1}‐t_{i}}$$ (1)

where $d\left(t_{i+1}\right)$ represents the displacement at time point $t_{i+1}$ , $d\left(t_{i}\right)$ represents the displacement at time point $t_{i}$ , $t_{i}$ represents the current time point, and $t_{i+1}$ represents the subsequent time point.

${GRF}_{X}$ **,** ${GRF}_{Y}$ , and ${GRF}_{Z}$ represent the ground reaction forces in the coronal, sagittal, and vertical axes respectively. BW represents the normalized body weight, and NDP represents the number of frames.

MATLAB R2022a was used to preprocess the surface EMG data and extract the muscle synergy patterns. Non-negative Matrix Factorization (NNMF) was employed to decompose the surface EMG signal envelope matrix (M) into a linear combination of a weight matrix ( $W_{i}$ , synergy structure) and a muscle activation coefficient matrix ( $C_{i}$ , activation coefficients) (Kim et al., 2017; Lee and Seung, 1999; Rabbi et al., 2020). As shown in Equations 2, 3.

$$M=\sum_{i=1}^{N}W_{i}C_{i}+e\;{\;W}_{i}\geq 0,{\;C}_{i}\geq 0$$ (2)

$$M^{\prime }=\sum_{i=1}^{N}W_{i}C_{i}\;W_{i}\geq 0,{\;C}_{i}\geq 0$$ (3)

M represents the original surface EMG signal envelope matrix with dimensions m × n, where m is the number of measured muscles, and n is the length of the time series of samples (i.e., the number of sampling points). N is the number of muscle synergies, $W_{i}$ represents the muscle synergy structure matrix with dimensions m × N, and Ci represents the muscle synergy activation coefficient matrix with dimensions N × n. e is the residual, and $M^{\prime }$ is the reconstructed EMG matrix obtained by multiplying $W_{i}$ and $C_{i}$ .

In muscle synergy extraction based on Non-negative Matrix Factorization, the number of muscles m and time series length n is known, but the number of muscle synergies N is unknown. Different values of N result in varying accuracies of the reconstructed EMG matrix $M^{\prime }$ , which is measured by the variability accounted for (VAF).

To determine the final number of synergies n during extraction, the Variance Accounted For (VAF) was used to evaluate the reconstruction accuracy of matrix $M^{\prime }$ relative to the original matrix M , as shown in Equation 4:

$$VAF=1-\frac{\sum_{i=1}^{m}\sum_{j=1}^{n}{\left(M\left(i,j\right)‐M^{\prime }\left(i,j\right)\right)}^{2}}{\sum_{i=1}^{m}\sum_{j=1}^{n}{\left(M\left(i,j\right)\right)}^{2}}$$ (4)

M is the original matrix and M′ is the reconstructed matrix. The VAF ranges from 0 to 1, with higher values indicating a higher reconstruction accuracy (i.e., M and M′ are closer).

The variance threshold method is used to determine the number of muscle synergies. Starting with N = 1, N increases incrementally during matrix decomposition. If the current number of synergies yielded an average VAF above all, the subjects’ synergy patterns were classified using k-means clustering analysis. The number of clusters k was determined by testing values from 1 to 10 and calculating the sum of squared errors (sumD) for each k. The process continued until sumD was minimally affected by the random initial centers of the k-means algorithm. Thus, the final number of clusters, k, in this study was set to four. a fixed value, the calculation stops and the number of muscle synergies is set to N. The synergy patterns of all subjects were classified using k-means clustering analysis. The number of clusters k was determined by testing values from 1 to 10, calculating the sum of squared errors (sumD) for each k. The process continued until the sumD was minimally affected by the random initial centers of the k-means algorithm. Thus, the final number of clusters, k, in this study was set to four. All synergy patterns were named Synergy 1-4, abbreviated as SYN1-4. According to the researchers’ studies, the active muscles in the synergistic module are defined as those with spatial component values exceeding 0.3 (Kubota et al., 2020; Milosevic et al., 2017). In this study, the activation curves for the leg thrusting action during each phase are divided into early, mid, and late phases based on time averaging.

Subsequently, Pearson’s correlation analysis was used to examine the relationship between the velocity of center of gravity and muscle synergies at each phase. Statistical significance was defined as *p* < 0.05, with “*” indicating *p* < 0.05 and “**” indicating *p* < 0.01. r > 0.7 is a strong positive correlation, 0.5 < r < 0.7 is a moderate positive correlation, and 0.3 < r < 0.5 is a weak positive correlation. r < −0.7 is a strong negative correlation, −0.5 > r > −0.7 is a moderate negative correlation, and −0.3 > r > −0.5 is a weak negative correlation. We selected the muscle synergies with the strongest correlation with the dynamic stability index at each stage as the best muscle synergies (r > 0.7 or r < −0.7).

### Phases of the leg stirrup movements

**FIGURE 2:**
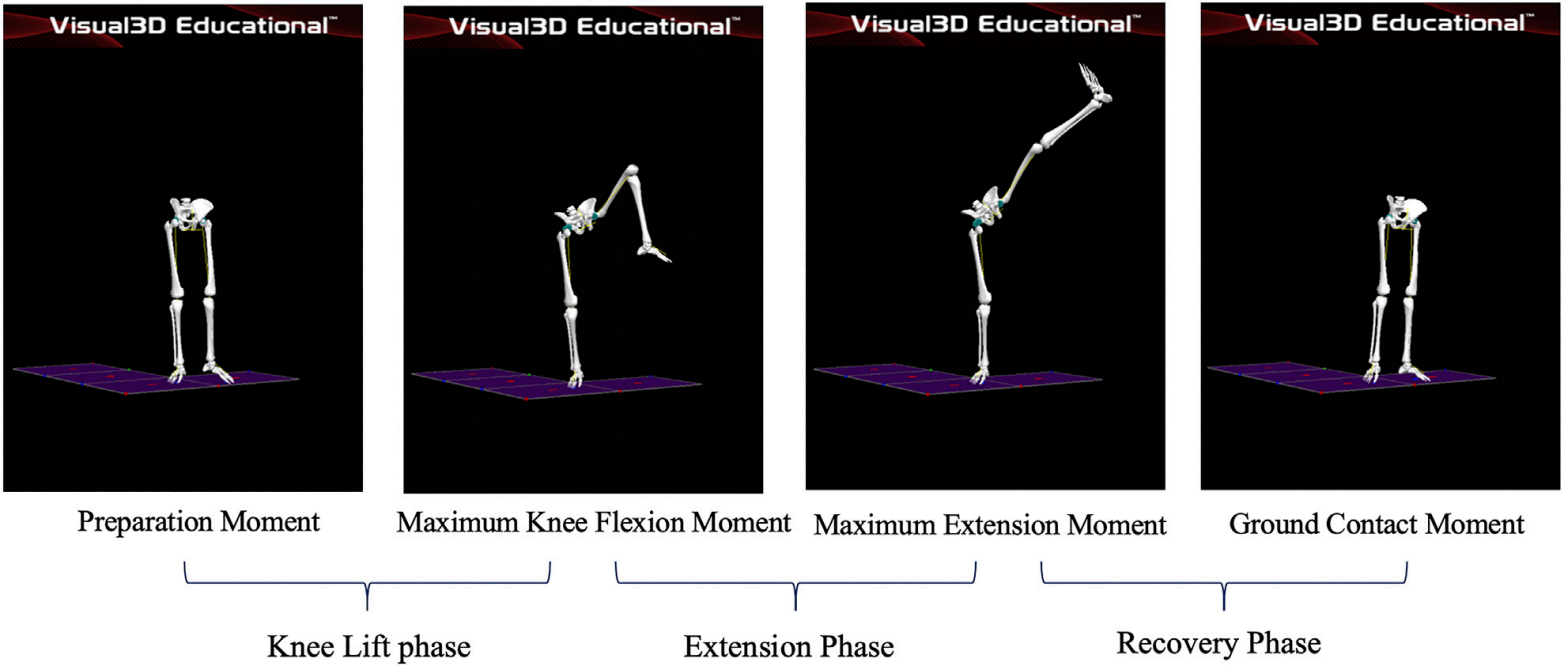
Schematic diagram of the phases of leg stirrup movements.

Preparation Moment: The moment the athlete’s left toe leaves the ground; Maximum Knee Flexion Moment: the moment the athlete’s left knee joint reaches maximum flexion; Maximum Extension Moment: the moment the lifted leg reaches maximum extension; Ground Contact Moment: the moment the athlete’s left toe touches the ground. Knee lift phase: This phase starts when the force line of the supporting leg on the force platform begins to decrease and ends when the knee joint of the supporting leg reaches maximum flexion. Extension phase: This phase starts when the knee joint of the stirrup leg reaches maximum flexion and ends when it reaches maximum extension. Recovery phase: The stirrup leg slowly flexes the knee and adducts the hip joint to land beside the supporting foot, with the heel touching the ground. The phase started when the knee joint of the stirrup leg reached its maximum extension and ended when the force line on the force platform returned to its initial value. As shown in Figure 2.

## Results

### Muscle synergy analysis of leg stirrup movements

**FIGURE 3:**
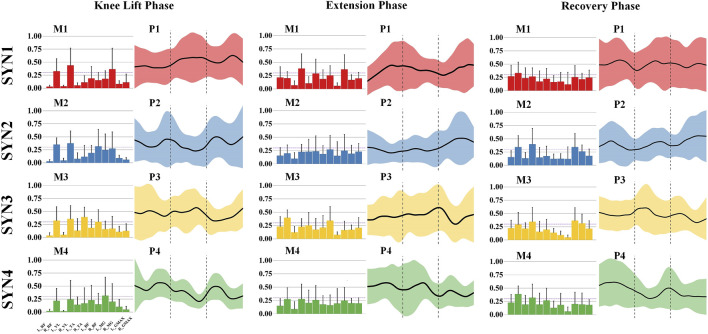
Activation weights and activation curves of leg stirrup movements. Note: In the bar chart on the left, the 12 measured muscles from left to right are: left rectus femoris (L_RF), right rectus femoris (R_RF), left vastus lateralis (L_VL), right vastus lateralis (R_VL), left tibialis anterior (L_TA), right tibialis anterior (R_TA), left biceps femoris (L_BF), right biceps femoris (R_BF), left medial gastrocnemius (L_MG), right medial gastrocnemius (R_MG), left gluteus maximus (L_GMAX), and right gluteus maximus (R_GMAX).

The kicking action is characterized by four muscle synergy patterns across three phases. In the knee-lift phase, the primary activated muscles in SYN1 are the right rectus femoris, right vastus lateralis, and right medial gastrocnemius, with the knee extensor muscles of the swing leg and the ankle plantar flexor muscles as the main activated muscle groups. These muscles are activated during the mid-to-late phase. In SYN2, the primary activated muscles are the right rectus femoris, right vastus lateralis, and right biceps femoris, with the knee extensor muscles of the swing leg and the stabilizing muscles of the stance leg as the main muscle groups, showing activity in both the early and late phases. SYN3 is dominated by the right rectus femoris, right vastus lateralis, and right tibialis anterior, with additional activation of the right biceps femoris. The knee extensor muscles of the swing leg are the main muscle group, with activation in the early-to-mid phase. SYN4’s primary activated muscle is the left medial gastrocnemius, with the plantar flexor muscles of the stance leg’s ankle joint being the main muscle group activated, showing activity in both the early and late phases. As shown in Figure 3.

During the extension phase, the main activated muscles in SYN1 are the right vastus lateralis and right medial gastrocnemius, primarily activating the knee extensor muscles of the swing leg, with activity in the early and late phases. In SYN3, the main activated muscles are the right rectus femoris and right biceps femoris, along with some activation of the left rectus femoris and left tibialis anterior, primarily activating the knee extensor muscles of the swing leg, with activity in the mid-to-late phase. SYN2 and SYN4 do not show significant activation of any major muscle groups during this phase. As shown in Figure 3.

In the recovery phase, the main activated muscle in SYN1 is the right rectus femoris, primarily activating the knee extensor muscles of the swing leg, with activity in the early and mid-to-late phases. SYN2’s main activated muscles are the right rectus femoris, right vastus lateralis, and right medial gastrocnemius, primarily activating the knee extensor muscles of the swing leg, with overall activity throughout the phase. In SYN3, the main activated muscles are the right rectus femoris, right vastus lateralis, right medial gastrocnemius, and left gluteus maximus, primarily activating the knee extensor muscles of the swing leg and the hip extensors of the stance leg, with activity in the mid-to-late phase. SYN4’s main activated muscles are the right vastus lateralis and right medial gastrocnemius, primarily activating the knee flexor muscles of the swing leg, with activity in the early phase. As shown in Figure 3.

### Correlation analysis of velocity of center of gravity with synergy patterns at each phase

During the knee lift phase, SYN1 showed a strong positive correlation with the VCG (r = 0.785**, *p* < 0.01), indicating that SYN1 provides good knee lift speed and stability, reducing wobble, and aiding smooth transitions. SYN2 showed a non-significant correlation with the VCG (r = 0.077, *p* > 0.05), indicating that SYN2 had little impact on dynamic and trunk stability at this phase. SYN3 and SYN4 showed moderate negative correlations with the VCG (r = −0.350**, *p* < 0.01; r = −0.551**, *p* < 0.01). This negative correlation may be related to improper muscle control or a lack of coordination between the hip and leg muscles. Improving the coordination of these muscles and techniques can reduce their negative impact on stability, enhance knee movement stability, and reduce body sway. As shown in Table 1.

**TABLE 1:** Correlation Results between Velocity of center of gravity and Synergy Patterns at Different Phases.

		SYN1		SYN2		SYN3		SYN4	
		r	p	r	p	r	p	r	P
VCG	Knee Lift Phase	0.785**	0.000	0.077	0.084	−0.350**	0.000	−0.551**	0.000
	Extension Phase	−0.508**	0.000	0.663**	0.000	−0.104*	0.000	−0.439**	0.000
	Recovery Phase	−0.129**	0.000	0.642**	0.000	−0.407**	0.000	−0.794**	0.000

Note: “*” and “**” indicate significant correlations between VCG, and muscle synergy.

During the extension phase, SYN1 showed a moderate negative correlation with the VCG (r = −0.508**, *p* < 0.01), indicating that excessive force or lack of coordination during extension may negatively impact dynamic and overall stability. SYN2 showed a strong positive correlation with the VCG (r = 0.663**, *p* < 0.01), indicating that the knee extensor muscle group positively affects the dynamic stability and stability of the Stirrup leg. Good SYN2 training helps stabilize extension movements and enhances the overall strength output stability. SYN3 and SYN4 showed negative correlations with the VCG (r = −0.104*, *p* < 0.01; r = −0.439**, *p* < 0.01). These negative correlations may be related to other control factors of the legs and hips, reflecting coordination or muscle imbalance. Improving muscle coordination related to these indicators can reduce wobble and instability caused by lack of coordination, ensuring stability during extension movements. As shown in Table 1.

During the recovery phase, SYN1 showed a weak negative correlation with the VCG (r = −0.129**, *p* < 0.01), indicating a weak but significant association between muscle activity recovery and VCG. This suggests that SYN1 has a certain inhibitory effect on VCG during the recovery process after knee lift. SYN2 showed a strong positive correlation with the VCG (r = 0.642**, *p* < 0.01), indicating that as VCG increases, SYN2 exhibits good muscle coordination during the recovery phase, reducing tremors and instability. SYN3 showed a moderate negative correlation with the VCG (r = −0.407**, *p* < 0.01), and SYN4 showed a strong negative correlation with VCG (r = −0.794**, *p* < 0.01). This indicates that SYN3 and SYN4 significantly contributed to instability during the recovery phase, suggesting the need to improve muscle endurance and posture control. As shown in Table 1.

## Discussion

The Leg Stirrup Movements in Tai Chi represent a complex skill that integrates precise control, balance, and coordination, significantly relying on the efficient interplay of the neuromuscular system. This movement necessitates rapid responses and powerful outputs from the lower limb muscles within a short timeframe, while also requiring optimal synergistic effects across the entire muscle groups involved in execution. During the knee-lifting phase, several muscles in the swinging leg, such as the rectus femoris and vastus lateralis, coordinate their actions, enabling the swinging leg to transition smoothly from a flexed position to gradual extension, thereby preparing for subsequent explosive force output. Stabilizing the center of mass is the first critical step in adjusting the Tai Chi posture, which is achieved by swiftly lowering the center of mass. This adjustment is a technical key to the right Leg Stirrup Movement in Tai Chi, enhancing the overall stability of the body. Muscle activation is not confined to the swinging leg; the hamstring group of the supporting leg and the calf muscles also contribute to force generation, thereby maintaining stability. The biceps femoris is primarily responsible for extending the hip joint, while the gastrocnemius assists in maintaining the balance of the center of mass by adjusting the ankle joint. This coordinated activation of multiple muscle groups ensures the smooth and effective transmission of the knee-lifting action (Law and Li, 2022; Chan et al., 2003). Throughout the knee-lifting process, the knee joint and ankle joint of the supporting leg remain relatively stable. In this phase, the single-leg support resembles an inverted pendulum, demonstrating uniaxial oscillation at the supporting leg’s ankle joint, with balance stabilization managed by the ankle strategy. Conversely, the supporting leg in the right Leg Stirrup Movement of Tai Chi utilizes a multiaxial oscillation pattern, incorporating hip flexion, knee flexion, and ankle flexion (Huang et al., 2009). Thus, this movement engages not only the ankle strategy but also requires the integration of the hip strategy to effectively control and coordinate body balance and stability, counteracting disturbances from the reaction forces generated during knee lifting (Van Der Burg et al., 2004). The ankle joint, knee joint, and hip joint of the supporting leg play pivotal roles during the leg stirrup process. The subtle adjustments made by these joints are essential for maintaining the center of mass within a stable range, thereby ensuring the fluidity of the knee-lifting action. Training in Tai Chi enhances practitioners’ ability to control their center of mass, thereby improving overall body balance through slow, deliberate, and precise movements (Wong et al., 2001).

The extension phase of the Leg Stirrup Movements in Tai Chi represents the pinnacle of force output within the entire movement. During this phase, the swinging leg rapidly transitions from a knee-lifting position to full extension, with explosive force being transferred from the leg muscles to the foot. The effectiveness of the extension movement hinges on the rapid extension of the knee joint and hip joint, with force generation dependent on the powerful contraction of the quadriceps and the synergistic activation of the hamstring group. Specifically, the rectus femoris and vastus lateralis play critical roles in achieving complete knee extension. Concurrently, the muscles of the supporting leg serve a stabilizing function during the extension phase, preventing excessive shifts in the center of mass due to the explosive release from the swinging leg. Successful execution of the extension movement necessitates precise coordination between the supporting and swinging legs, ensuring that the center of mass remains stable while generating explosive force (Henni et al., 2020). Central to the extension movement is the formation of the lower limb kinetic chain and the generation of torque. The synergistic activation of leg muscles and the movement of joints are crucial in forming this kinetic chain. Consequently, force is sequentially transferred from the hip to the knee and ultimately to the foot, resulting in a complete torque output (Fort-Vanmeerhaeghe et al., 2016). The rapid contraction of the quadriceps facilitates knee extension, while subtle adjustments at the ankle joint ensure the effective transmission of torque. The efficient utilization of force transmission and the kinetic chain in Tai Chi maximizes overall force output (Wu, 2012; Tseng et al., 2007). During the extension phase, the body’s center of mass undergoes rapid shifts. At this juncture, fine adjustments to the knee joint and hip joint of the supporting leg are essential for maintaining stability. Upon completion of the extension by the swinging leg, the adjustments made at the ankle joint and hip joint of the supporting leg become particularly critical, as they mitigate the risk of imbalance resulting from the explosive force release (Myer et al., 2008). The integration of the ankle strategy and hip strategy is vital at this stage. The ankle strategy regulates the body’s anterior-posterior balance through the flexibility of the ankle joint, while the hip strategy ensures lateral stability via adjustments at the hip joint. This combined control mechanism effectively enables the supporting leg to counteract greater torque-induced disturbances to balance (Boonstra et al., 2013).

The recovery phase mainly involved returning the leg from the extended position to the initial position. A correct and smooth recovery phase provides a smooth transition for continuous action, avoiding unnecessary power loss and potential injury risks. In addition to requiring control and coordination, good muscle elasticity and recoil ability are required to ensure the continuity and efficiency of movements (Eden et al., 2022). In this study, during the extension phase, the four muscle synergy patterns mainly involved the rectus femoris, vastus lateralis, and medial gastrocnemius of the swinging leg, which were active during the early phase. In Tai Chi, the contraction patterns of the muscles continuously change with movement changes (Gouglidis et al., 2010). This continuous change in muscle contraction patterns may explain why Tai Chi improves lower limb strength. During Tai Chi practice, the way muscles contract varies according to different Tai Chi movements. Continuous changes in movement require different muscle strengths to maintain postural stability. This relatively complex and diverse exercise improves the cooperation between the agonist and antagonist muscles, enhancing muscle strength and control. Through highly coordinated control, it ensures smooth transitions and precise execution of movements, especially in maintaining balance during rapid posture changes (Yiou et al., 2017). Precise muscle control is necessary for complex recovery movements, particularly to maintain postural stability when quick and accurate recovery is required. The strong positive correlation between SYN2 and the VCG indicates that as VCG increases, the activity of recovery muscle groups also increases significantly. This indicates that maintaining control and balance during rapid center of gravity shifts requires more muscle involvement. Rapid and precise muscle responses are needed to adjust posture and prevent instability-induced imbalance during quick center-of-gravity shifts (Anderson and Behm, 2005). The strong negative correlation of SYN4 indicates that the muscle activity required for recovery significantly decreases during rapid center-of-gravity shifts. This reflects high movement efficiency, where practitioners can effectively use momentum and coordination to complete recovery actions.

This study explored the neuromuscular activity characteristics of Tai Chi athletes during Leg Stirrup Movements and identified the optimal muscle synergy patterns at various phases. However, the study has some limitations. First, since all participants were Tai Chi athletes with sports rankings, the generalizability of the findings is limited and cannot be extended to a broader population. Second, the study did not incorporate multiple indicators to comprehensively assess the technical level and effectiveness of Tai Chi. Lastly, although the study identified the optimal synergy patterns at different phases, it did not analyze the underlying mechanisms, which will be further explored in future research.

## Conclusion

In the Leg Stirrup Movements of Tai Chi, the optimal muscle synergy patterns at different stages play a critical role in determining the movement’s balance, force output, and technical performance. This study identifies the optimal muscle synergy pattern during the knee-lifting phase as SYN1. This pattern ensures the stability and fluidity of leg movements by coordinating the activation of the knee extensors. In contrast, during the extension and recovery phases, SYN2 is regarded as the most effective pattern, significantly improving dynamic balance and force output efficiency in the leg. By optimizing these key muscle synergy stages, athletes can more effectively engage their muscle groups, thereby enhancing movement coordination, technical expressiveness, and overall stability. Consequently, training programs should emphasize improving SYN1’s coordination during the knee-lifting phase and enhancing SYN2’s force output and coordination during the extension and recovery phases. This focus will maximize the overall performance of the Leg Stirrup Movements in Tai Chi.

## Data Availability

The raw data supporting the conclusions of this article will be made available by the authors, without undue reservation.
